# Author Correction: Mitochondrial calcium uniporter stabilization preserves energetic homeostasis during Complex I impairment

**DOI:** 10.1038/s41467-022-31304-5

**Published:** 2022-06-20

**Authors:** Enrique Balderas, David R. Eberhardt, Sandra Lee, John M. Pleinis, Salah Sommakia, Anthony M. Balynas, Xue Yin, Mitchell C. Parker, Colin T. Maguire, Scott Cho, Marta W. Szulik, Anna Bakhtina, Ryan D. Bia, Marisa W. Friederich, Timothy M. Locke, Johan L. K. Van Hove, Stavros G. Drakos, Yasemin Sancak, Martin Tristani-Firouzi, Sarah Franklin, Aylin R. Rodan, Dipayan Chaudhuri

**Affiliations:** 1 Nora Eccles Harrison Cardiovascular Research and Training Institute, University of Utah, Salt Lake City, UT USA; 2 Molecular Medicine Program, University of Utah, Salt Lake City, UT USA; 3 University of Nevada Reno School of Medicine, Reno, NV USA; 4 Clinical & Translational Science Institute, University of Utah, Salt Lake City, UT USA; 5 Section of Clinical Genetics and Metabolism, Department of Pediatrics, University of Colorado, Aurora, CO USA; 6 Department of Pathology and Laboratory Medicine, Children’s Hospital Colorado, Aurora, CO USA; 7 Department of Pharmacology, University of Washington, Seattle, WA USA; 8 Division of Cardiovascular Medicine, Department of Internal Medicine, University of Utah, Salt Lake City, UT USA; 9 Division of Pediatric Cardiology, University of Utah School of Medicine, Salt Lake City, UT USA; 10 Division of Nephrology and Hypertension, Department of Internal Medicine, University of Utah, Salt Lake City, UT USA; 11 Department of Biochemistry, Department of Biomedical Engineering, University of Utah, Salt Lake City, UT USA

**Keywords:** Energy metabolism, Cardiomyopathies, Mechanisms of disease, Calcium signalling, Proteomic analysis

Correction to: *Nature Communications* 10.1038/s41467-022-30236-4, published online 19 May 2022.

The original version of this Article contained an error in Acknowledgements, which incorrectly read ‘We thank Edward Owusu-Ansah, Alex Whitworth, Ronald Davis, and the Bloomington Drosophila Stock Center at Indiana University (supported by NIH P40OD018537) for fly stocks, Michael Ryan for the NDUFB10KO, NDUFS4KO, and control cell lines, David Clapham for other HEK293T cell lines, John Elrod, Ronald Kahn, and Nils-Göran Larsson for mouse lines, James Marvin and Flow Cytometry Core staff at the University of Utah (supported by NIH P30CA042014), Small Animal Ultrasound Core staff at the University of Utah, and Derek Warner and DNA Sequencing Core staff at the University of Utah.’ The correct version states ‘We thank Edward Owusu-Ansah, Alex Whitworth, Ronald Davis, and the Bloomington Drosophila Stock Center at Indiana University (supported by NIH P40OD018537) for fly stocks, Vamsi Mootha for multiple plasmids and MCU knockout cell lines, Michael Ryan for the NDUFB10KO, NDUFS4KO, and control cell lines, David Clapham for other HEK293T cell lines, John Elrod, Ronald Kahn, and Nils-Göran Larsson for mouse lines, James Marvin and Flow Cytometry Core staff at the University of Utah (supported by NIH P30CA042014), Small Animal Ultrasound Core staff at the University of Utah, and Derek Warner and DNA Sequencing Core staff at the University of Utah’. This has been corrected in both the PDF and HTML versions of the Article.

