# Supplementary material for: Mitochondrial calcium uniporter stabilization preserves energetic homeostasis during Complex I impairment
Source: Nat Commun. 2022 May 19;13:2769. doi: 10.1038/s41467-022-30236-4 (PMC9120069; doi:10.1038/s41467-022-30236-4)
Supplement: Supplementary file 1 — Supplementary Information [file 41467_2022_30236_MOESM1_ESM.pdf]

Supplementary Table 1. *Drosophila* genotypes.

Abbreviated Genotype	Female Genotype	Male Genotype
+/+	*w/w Berlin*	*w/Y Berlin*
+/B10^{RNAi} MCU^{DQEQ}	*w/yw; +/UAS-NDUFB10 RNAi*$^{TRiP-Bl29592}$ *UAS-MCU*^{D206Q,E263Q}	*w/Y; +/UAS-NDUFB10 RNAi*$^{TRiP-Bl29592}$ *UAS-MCU*^{D206Q,E263Q}
MHC>	*w/w; MHC-GAL4/+*	*w/Y; MHC-GAL4/+*
MHC>MCU^{DQEQ}	*w/w; MHC-GAL4/UAS-MCU*^{D206Q,E263Q}	*w/Y; MHC-GAL4/UAS-MCU*^{D206Q,E263Q}
MHC>B10^{RNAi}	*w/yw; MHC-GAL4/UAS-NDUFB10 RNAi*$^{TRiP-Bl29592}$	*w/Y; MHC-GAL4/UAS-NDUFB10 RNAi*$^{TRiP-Bl29592}$
MHC>B10^{RNAi} MCU^{DQEQ}	*w/yw; MHC-GAL4/UAS-NDUFB10 RNAi*$^{TRiP-Bl29592}$ *UAS-MCU*^{D206Q,E263Q}	*w/Y; MHC-GAL4/UAS-NDUFB10 RNAi*$^{TRiP-Bl29592}$ *UAS-MCU*^{D206Q,E263Q}
+/MCU^{1} B10^{RNAi}	*w/yw; +/MCU*^{1} *UAS-NDUFB10 RNAi*$^{TRiP-Bl29592}$	*w/Y; +/MCU*^{1} *UAS-NDUFB10 RNAi*$^{TRiP-Bl29592}$
MCU^{1}/MCU^{1}	*w/w; MCU*^{1}*/MCU*^{1}	*w/Y; MCU*^{1}*/MCU*^{1}
MHC>B10^{RNAi} MCU^{1}/MCU^{1}	*w/yw; MHC-GAL4 MCU*^{1}*/MCU*^{1} *UAS-NDUFB10 RNAi*$^{TRiP-Bl29592}$	*w/Y; MHC-GAL4 MCU*^{1}*/MCU*^{1} *UAS-NDUFB10 RNAi*$^{TRiP-Bl29592}$
+/A13^{RNAi} MCU^{DQEQ}	*w/yw; +/UAS-NDUFA13 RNAi*$^{TRiP-Bl43279}$ *UAS-MCU*^{D206Q,E263Q}	*w/Y; +/UAS-NDUFA13 RNAi*$^{TRiP-Bl43279}$ *UAS-MCU*^{D206Q,E263Q}
MHC>A13^{RNAi}	*w/yw; MHC-GAL4/UAS-NDUFA13 RNAi*$^{TRiP-Bl43279}$	*w/Y; MHC-GAL4/UAS-NDUFA13 RNAi*$^{TRiP-Bl43279}$
MHC>A13^{RNAi} MCU^{DQEQ}	*w/yw; MHC-GAL4/UAS-NDUFA13 RNAi*$^{TRiP-Bl43279}$ *UAS-MCU*^{D206Q,E263Q}	*w/Y; MHC-GAL4/UAS-NDUFA13 RNAi*$^{TRiP-Bl43279}$ *UAS-MCU*^{D206Q,E263Q}
MHC>MCU^{WT} B10^{RNAi} MCU^{1}/MCU^{1}	*w/w; UAS-MCU*^{WT}*/+; MCU*^{1} *MHC-GAL4/MCU*^{1} *UAS-NDUFB10 RNAi*$^{TRiP-Bl29592}$	*w/Y; UAS-MCU*^{WT}*/+; MCU*^{1} *MHC-GAL4/MCU*^{1} *UAS-NDUFB10 RNAi*$^{TRiP-Bl29592}$
MHC>MCU$^{\Delta NTD}$ B10^{RNAi} MCU^{1}/MCU^{1}	*w/w; UAS-MCU*$^{\Delta NTD}$*/+; MCU*^{1} *MHC-GAL4/MCU*^{1} *UAS-NDUFB10 RNAi*$^{TRiP-Bl29592}$	*w/Y; UAS-MCU*$^{\Delta NTD}$*/+; MCU*^{1} *MHC-GAL4/MCU*^{1} *UAS-NDUFB10 RNAi*$^{TRiP-Bl29592}$
da>MCU$^{\Delta NTD}$ MCU^{1}/MCU^{1}	*w/ w; UAS-MCU*$^{\Delta NTD}$*/+; MCU*^{1} *da-GAL4/MCU*^{1}	*w/Y; UAS-MCU*$^{\Delta NTD}$*/+; MCU*^{1} *da-GAL4/MCU*^{1}
+/B10^{RNAi} MCU^{NTD}	*w/yw; UAS-MCU*^{NTD}*/+; UAS-NDUFB10 RNAi*$^{TRiP-Bl29592}$	*w/Y UAS-MCU*^{NTD}*/+; UAS-NDUFB10 RNAi*$^{TRiP-Bl29592}$
da>MCU^{NTD}	*w/w; UAS-MCU*^{NTD}*/+; da-GAL4*	*w/Y; UAS-MCU*^{NTD}*/+; da-GAL4*
MHC> MCU^{NTD} B10^{RNAi}	*w/yw; UAS-MCU*^{NTD}*/+; MHC-GAL4/UAS-NDUFB10 RNAi*$^{TRiP-Bl29592}$	*w/Y; UAS-MCU*^{NTD}*/+; MHC-GAL4/UAS-NDUFB10 RNAi*$^{TRiP-Bl29593}$

Supplementary Table 2. Primers

Gene	Sequence
Human MCU	ACGGTACACCAGAGGATCGC<br>TGAGTGTGAACTGACAGCGTT
Human EMRE	CGGTGAAAATGTCCTTCGGC<br>CGGTCATGGACCATACAGGA
Human MICU1	GGACAGTGGCTAAAGTGGAGC<br>CATGAGGCGAGTGAAACCC

Human GAPDH	TGTTGCCATCAATGACCCCTT<br>CTCCACGACGTACTCAGCG
Mouse MCU	AACTGCAAGAGGAGGATCGG<br>GTCTCTCTTTGGTGGCCGTA
Mouse TFAM	CAGGAGGCAAAGGATGATTC<br>CCAAGACTTCATTTCATTGTCG
Mouse GAPDH	TGCACCACCAACTGCTTAG<br>GATGCAGGGATGATGTTC

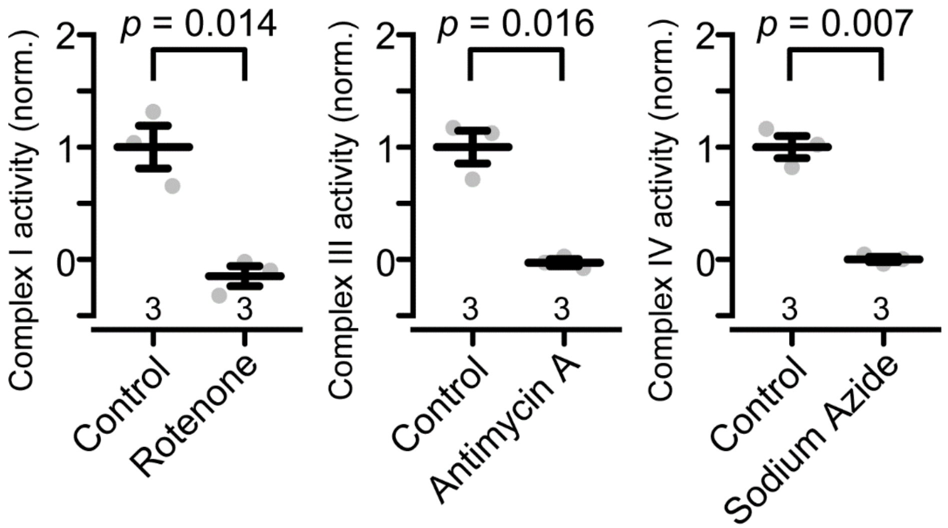

Supplementary Figure 1. Chronic pharmacological inhibition suppresses ETC activity. HEK293T cells grown in media containing either Complex I (1 μM rotenone), III (1 μM antimycin A), or IV (200 μM sodium azide) inhibitors for 2-3 days. Activity of the corresponding complex was subsequently determined using NADH oxidation (Complex I), cytochrome C reduction (Complex III), or cytochrome C oxidation (Complex IV) in mitochondrial samples of equivalent citrate synthase activity. Activity is normalized (norm.) to the average of vehicle-treated control cells. Summary data are presented as mean values ± SEM. Statistics: two-sided Student's *t*-test. Source data are provided as a Source Data file.

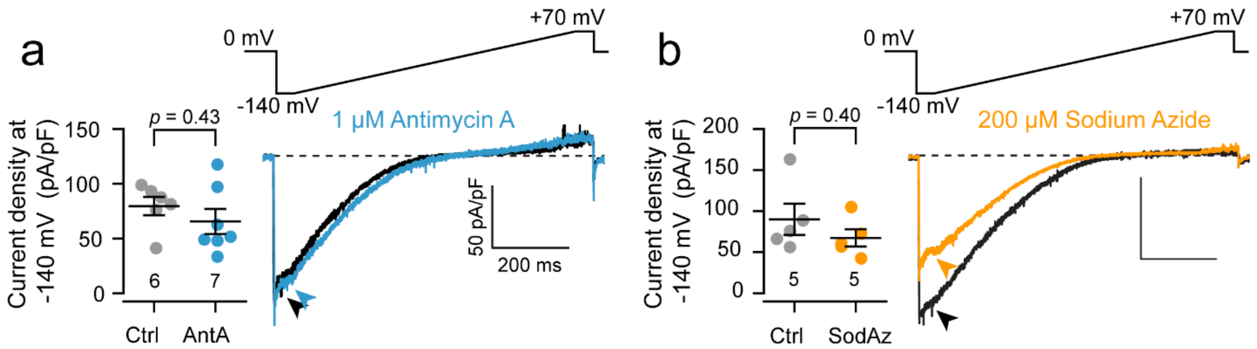

Supplementary Figure 2. Inhibition of Complex III or IV does not alter uniporter currents. A-B. Left, summary data, showing magnitude of current density at -140 mV (arrowheads in exemplars). Right, exemplar I_{MiCa} traces, with voltage ramp protocol depicted above. **A.** Inhibition of Complex III with 1 μM antimycin A does not alter I_{MiCa}. **B.** Inhibition of Complex IV with 200 μM sodium azide does not alter I_{MiCa}. Statistics: two-sided Mann-Whitney U test. Summary data are presented as mean values ± SEM. Source data are provided as a Source Data file.

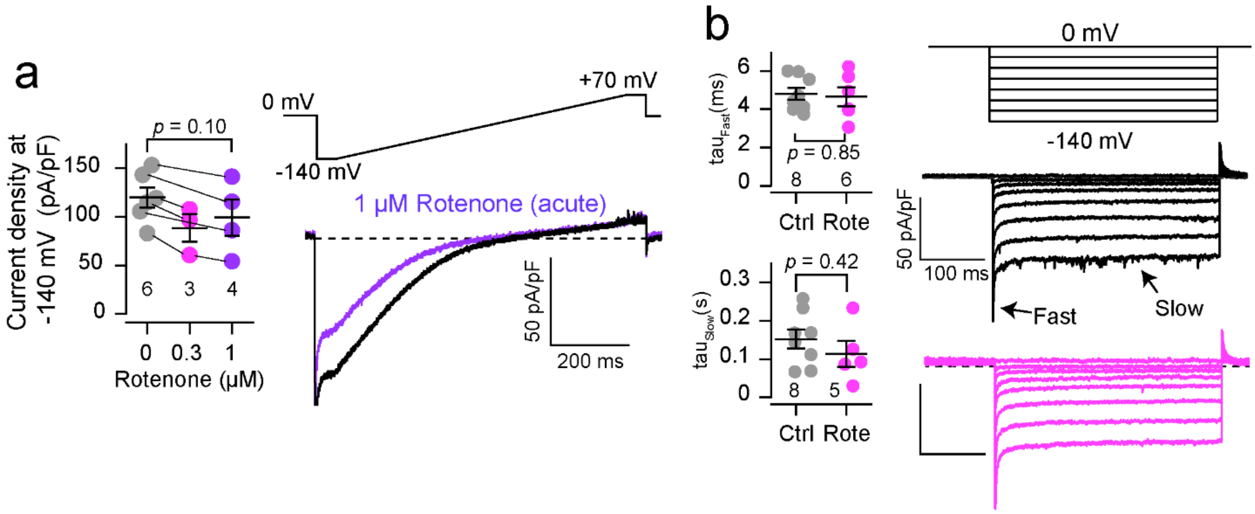

Supplementary Figure 3. Uniporter enhancement is not an off-target effect of rotenone. A. Acute addition of 1 µM rotenone during electrophysiological recordings does not increase I_{MiCa}. Lines connect individual trials before and after rotenone addition. **B.** Chronic Complex I inhibition with rotenone does not alter I_{MiCa} fast or slow inactivation kinetics. Statistics: (A) two-sided Wilcoxon signed-ranks test; (B) two-sided Mann Whitney U test. Summary data are presented as mean values ± SEM. Source data are provided as a Source Data file.

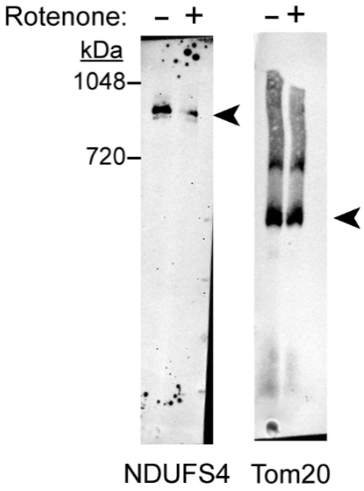

Supplementary Figure 4. Chronic rotenone treatment reduces Complex I levels. HEK293T mitochondria were solubilized in 1% digitonin and 10 µg protein analyzed via blue-native polyacrylamide gel electrophoresis (BN-PAGE) and immunoblotting as shown. Identical amounts were loaded in separate wells and TOM20 was used as loading control. Arrowheads point to NDUFS4 or TOM20 in the respective blots. Source data are provided as a Source Data file.

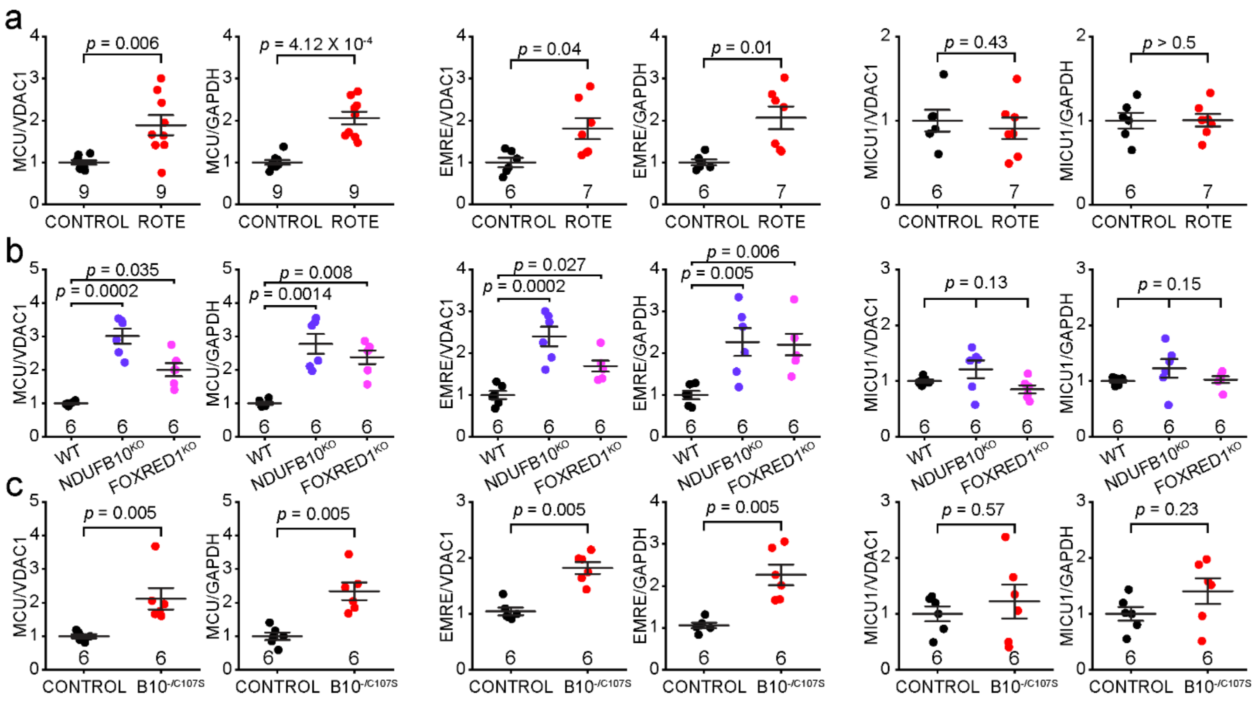

Supplementary Figure 5. Quantification of western blots in Figure 1c. The ratio of intensities relative to loading controls (GAPDH or VDAC1) were normalized to the average of the vehicle-treated or wild-type cells. Condition are control versus rotenone treatment (**A**); wild-type, FOXRED1^{KO}, and NDUFB10^{KO} cells (**B**); and control versus NDUFB10$^{-/C107S}$ IPSCs (**C**). Summary data are presented as mean values ± SEM. Data collected from separate gels due to number of samples, each containing all conditions and processed in parallel. Statistics: (A, C) two-sided Mann-Whitney U tests; (B) Kruskal-Wallis followed by Dunn's test with Bonferroni correction. Source data are provided as a Source Data file.

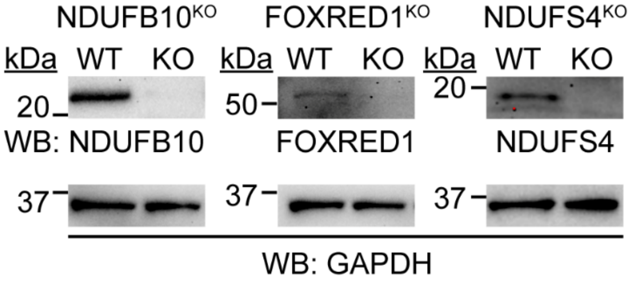

Supplementary Figure 6. NDUFB10, FOXRED1, and NDUFS4 protein depletion in NDUFB10^{KO}, FOXRED1^{KO}, and NDUFS4^{KO} cells, assessed by Western blot. Images are representative of 2 trials. Source data are provided as a Source Data file.

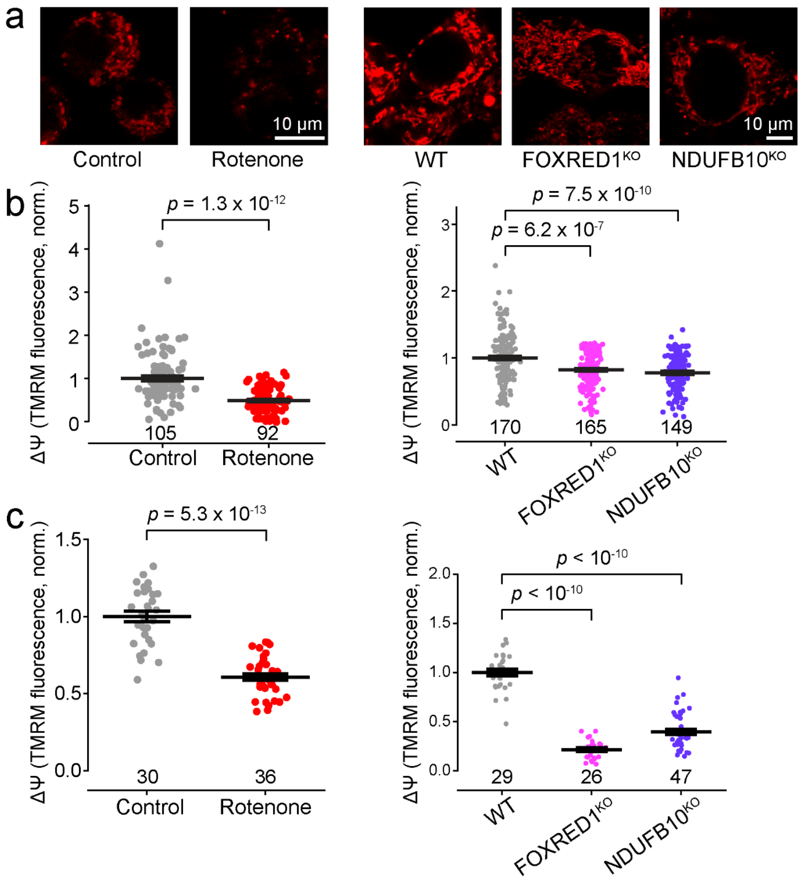

Supplementary Figure 7. Complex I inhibition depolarizes ΔΨ. Intact cells loaded with 20 nM tetramethyl rhodamine methyl ester (TMRM). Each point is an individual cell, and values are normalized to the average of the control/WT levels. **A.** Representative images. **B.** In phosphate-buffered saline TMRM fluorescence was diminished by 52% for rotenone, 16% for FOXRED1^{KO}, and 20% for NDUFB10^{KO}. **C.** In high-potassium buffer to depolarize the plasma membrane, TMRM diminished by 40% for rotenone, 79% for FOXRED1^{KO}, and 61% for NDUFB10^{KO}. Note that different batches of cells were used in (B) and (C). Summary data are presented as mean values ± SEM. Statistics: (Left) two-sided Student's *t*-test; (Right) 1-way ANOVA followed by Bonferroni-corrected means comparisons. Source data are provided as a Source Data file.

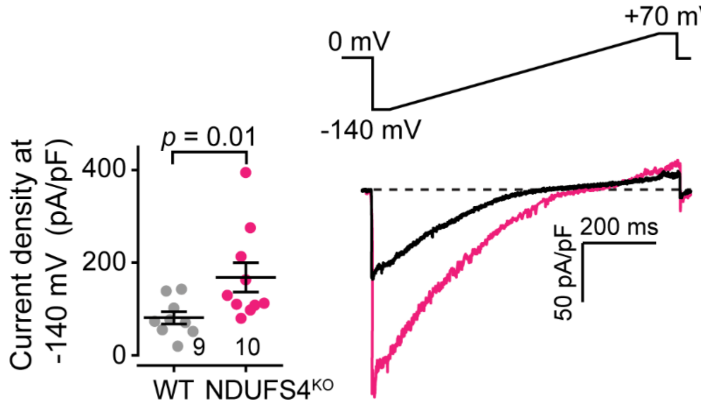

Supplementary Figure 8. Enhanced I_{MiCa} in NDUFS4^{KO} cells. Summary data are presented as mean values ± SEM. Statistics: two-sided Mann-Whitney U test. Source data are provided as a Source Data file.

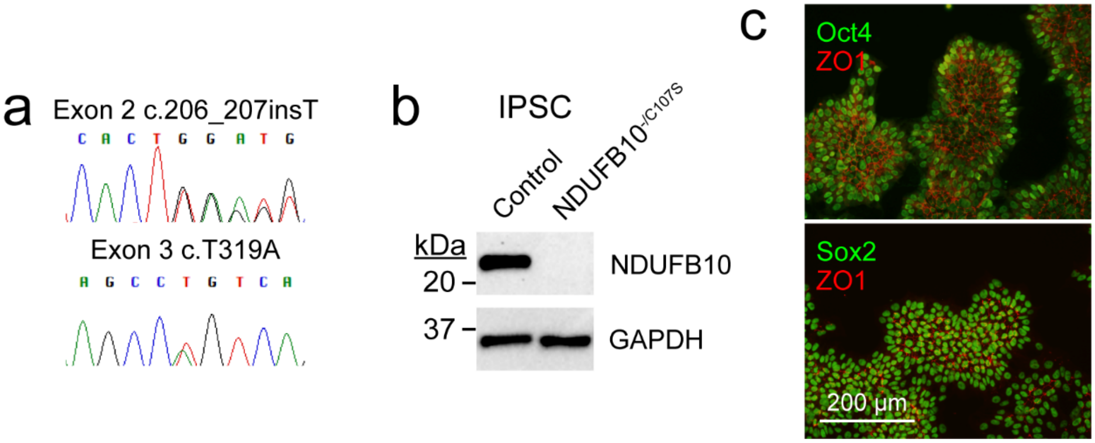

Supplementary Figure 9. Patient-derived IPSCs with NDUFB10 mutations. A. NDUFB10 inactivating mutations are preserved in patient-derived IPSCs. **B.** NDUFB10 protein depletion in NDUFB10$^{-/C107S}$ IPSCs. **C.** Confirmation that stem cell markers are expressed in NDUFB10$^{-/C107S}$ IPSCs. Images are representative of 2 trials (B, C). Source data are provided as a Source Data file.

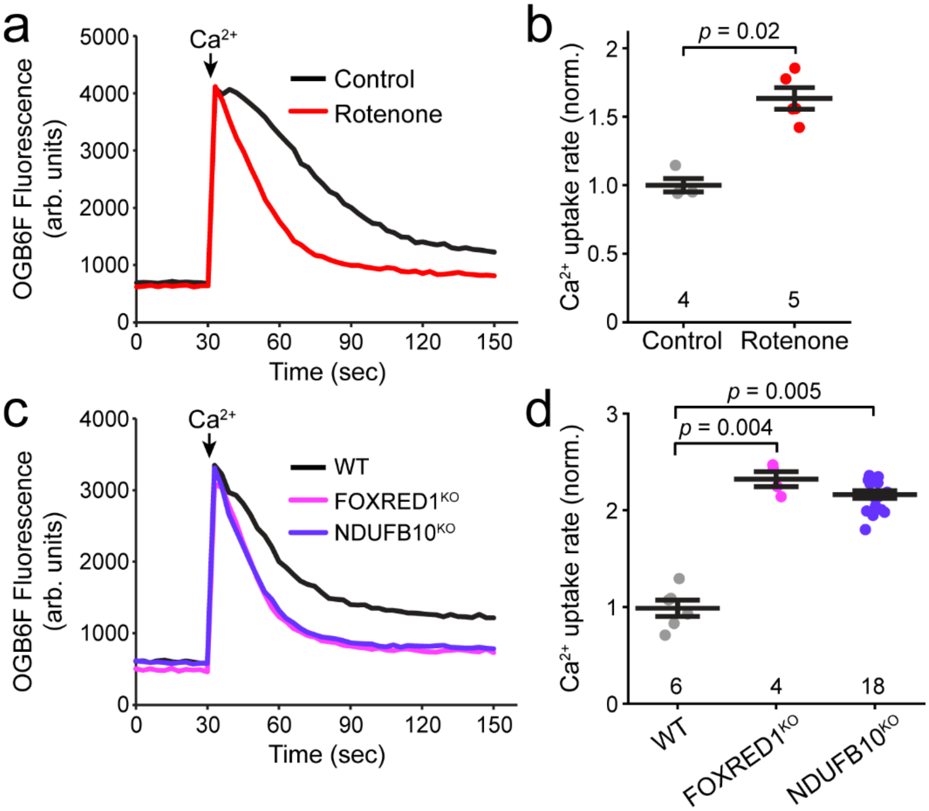

Supplementary Figure 10. Mitochondrial Ca^{2+} uptake is increased during Complex I inhibition. A, C. Exemplar traces of Ca^{2+} uptake in permeabilized cells. Following a 25 µM Ca^{2+} pulse (arrow), Ca^{2+} uptake into mitochondria is reflected as a gradual fall in Oregon Green BAPTA 6F (OGB6F) fluorescence. Arb. units, arbitrary units. **B, D.** Summary of Ca^{2+} uptake rates during the first 30 seconds after the Ca^{2+} pulse, normalized to the average of the controls. Summary data are presented as mean values ± SEM. Statistics: (B) two-sided Mann-Whitney U test; (Right) Kruskal-Wallis followed by Dunn's test with Bonferroni correction. Source data are provided as a Source Data file.

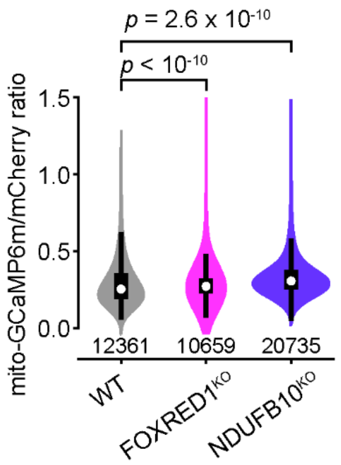

Supplementary Figure 11. Increased mitochondrial Ca^{2+} levels in NDUFB10^{KO} and FOXRED1^{KO} cells. The data corresponds to 206 nM, 221 nM, and 370 nM average free mitochondrial [Ca^{2+}] for WT, FOXRED1^{KO}, and NDUFB10^{KO} cells. Violin plot insets display Tukey boxplots. Statistics: 1-way ANOVA followed by Bonferroni-corrected means comparisons. Source data are provided as a Source Data file.

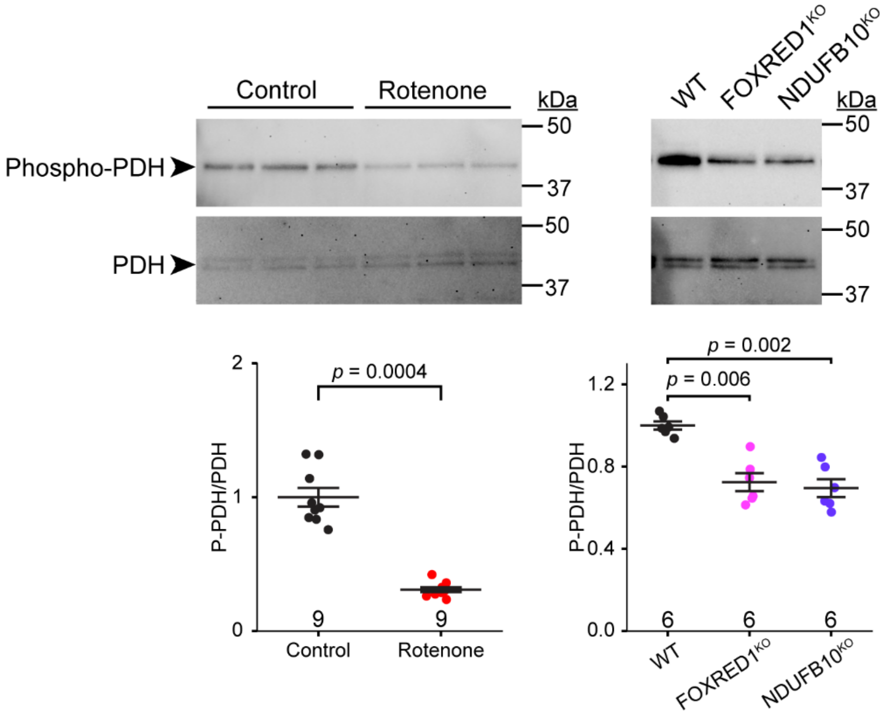

Supplementary Figure 12. Reduced PDH phosphorylation in Complex I deficient cells. Top, Western blots for phosphor-PDH and PDH. Bottom, corresponding densitometry of phospho-PDH/PDH ratio, normalized to average of ratios in control cells. Summary data are presented as mean values ± SEM. Data collected from separate gels due to number of samples, each containing all conditions and processed in parallel. Statistics: (Left) two-sided Mann-Whitney U test; (Right) Kruskal-Wallis followed by Dunn's test with Bonferroni correction. Source data are provided as a Source Data file.

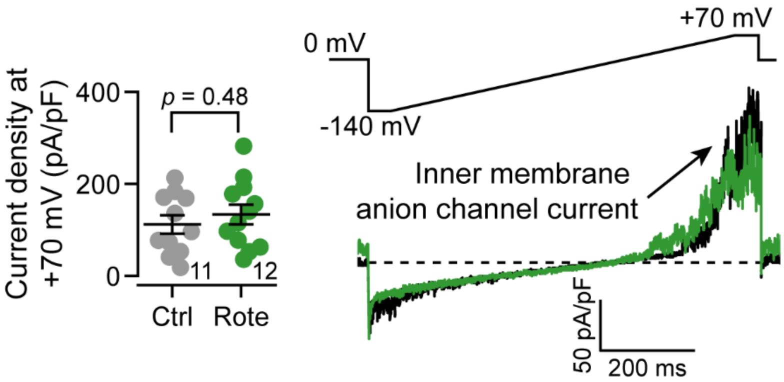

Supplementary Figure 13. Inner membrane anion channel currents are unaltered by chronic rotenone treatment. Each point in the summary graph shows current density at +80 mV, indicated by the arrow on the exemplar at the right. Summary data are presented as mean values ± SEM. Statistics: two-sided Mann-Whitney U test. Source data are provided as a Source Data file.

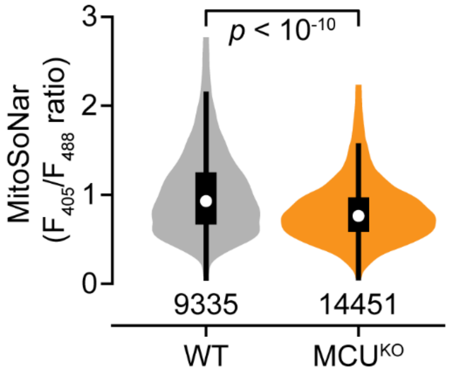

Supplementary Figure 14. NADH/NAD$^+$ ratio is diminished in MCU^{KO} cells. There is a 20% reduction in the MitoSoNar ratio in MCU^{KO} cells compared to wild-type. Violin plot insets display Tukey boxplots. Statistics: two-sided Student's *t*-test. Source data are provided as a Source Data file.

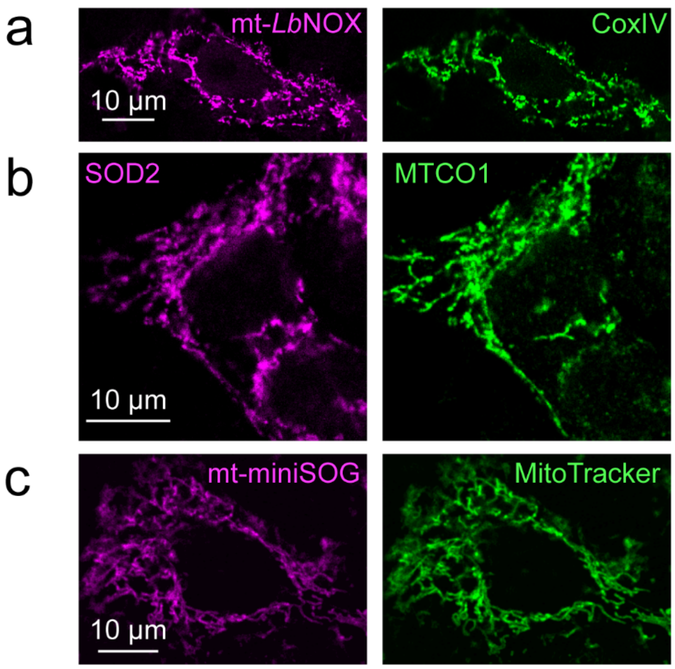

Supplementary Figure 15. Mitochondrially-targeted constructs. A. Immunocytochemistry shows mitochondrial targeting of mt-*Lb*NOX (left). CoxIV antibody labels mitochondria. **B.** Immunocytochemistry of SOD2 overexpression. MTCO1 antibody labels mitochondria. **C.** Mito-miniSOG fluorescence in live cells. MitoTracker labels mitochondria. Images are representative of 2 trials.

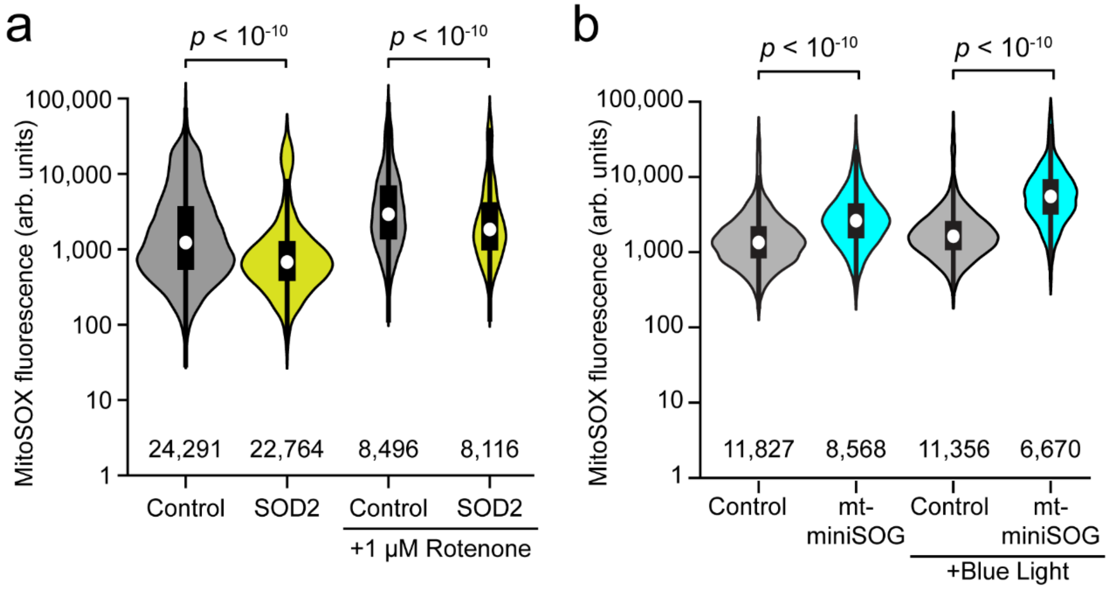

Supplementary Figure 16. Modulation of mitochondrial ROS production. A. SOD2 overexpression blunts ROS. Control or SOD2-expressing cells were labeled with MitoSOX and fluorescence intensity measured by flow cytometry. **B.** Blue light activation of mitochondrial miniSOG expression enhances ROS production. Control or mt-miniSOG expressing cells were labeled with MitoSOX and exposed to blue light for 10 minutes prior to flow cytometric analysis. Violin plot insets display Tukey boxplots. Statistics: 1-way ANOVA followed by Bonferroni-corrected means comparisons. Source data are provided as a Source Data file.

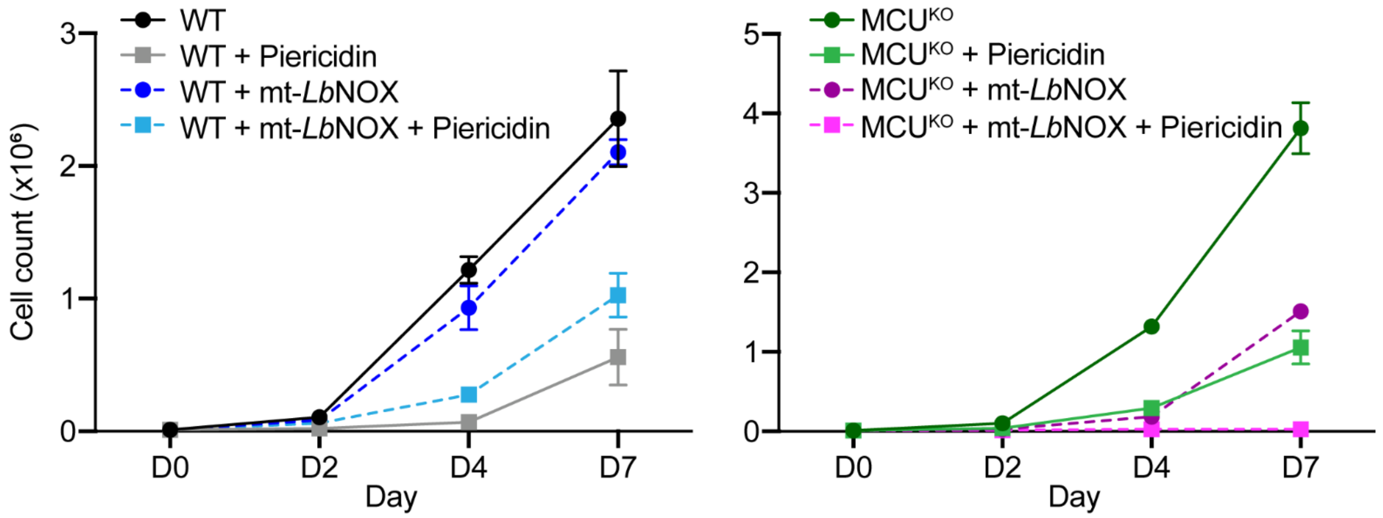

Supplementary Figure 17. Uniporter activity is necessary for the rescue of Complex I dysfunction by mt-*Lb*NOX. Piericidin incubation blunts cell proliferation in both WT (left) and MCU^{KO} (right) cells. Mt-*Lb*NOX expression rescues the cell proliferation defect in WT but not MCU^{KO} cells. N = 3 replicates. Summary data are presented as mean values ± SEM. Source data are provided as a Source Data file.

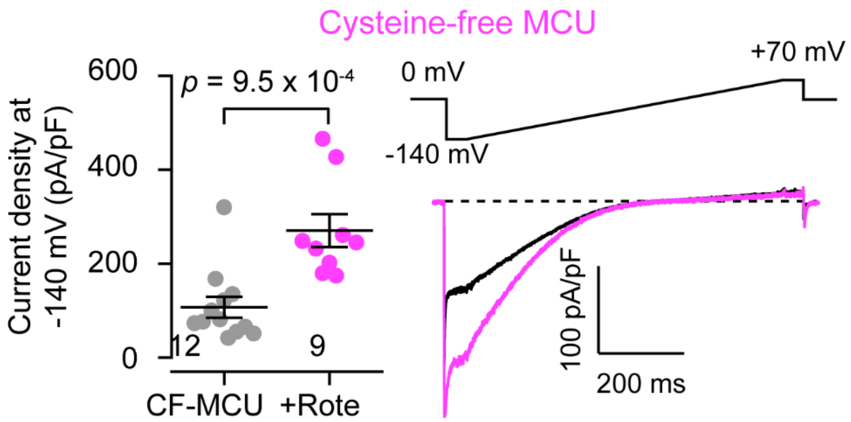

Supplementary Figure 18. I_{MiCa} enhancement persists after Complex I impairment in MCU^{KO} cells expressing cysteine-free MCU (CF-MCU-Flag). Summary data are presented as mean values ± SEM. Statistics: two-sided Mann-Whitney U test. Source data are provided as a Source Data file.

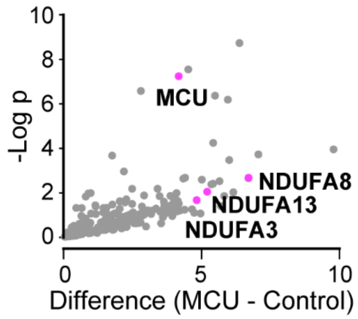

Supplementary Figure 19. Complex I proteins detected in MCU-Flag proteomic screen. Source data are provided as a Source Data file.

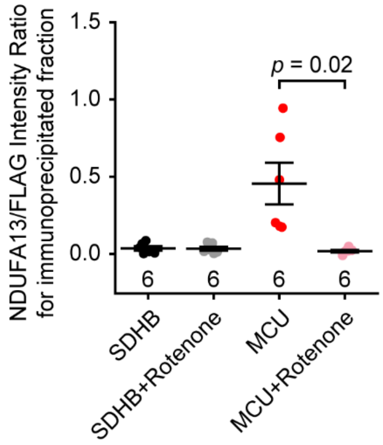

Supplementary Figure 20. Quantification of NDUFA13-MCU-Flag co-immunoprecipitation. Data are presented as mean values ± SEM. Statistics: Kruskal-Wallis followed by Dunn's test with Bonferroni correction. Source data are provided as a Source Data file.

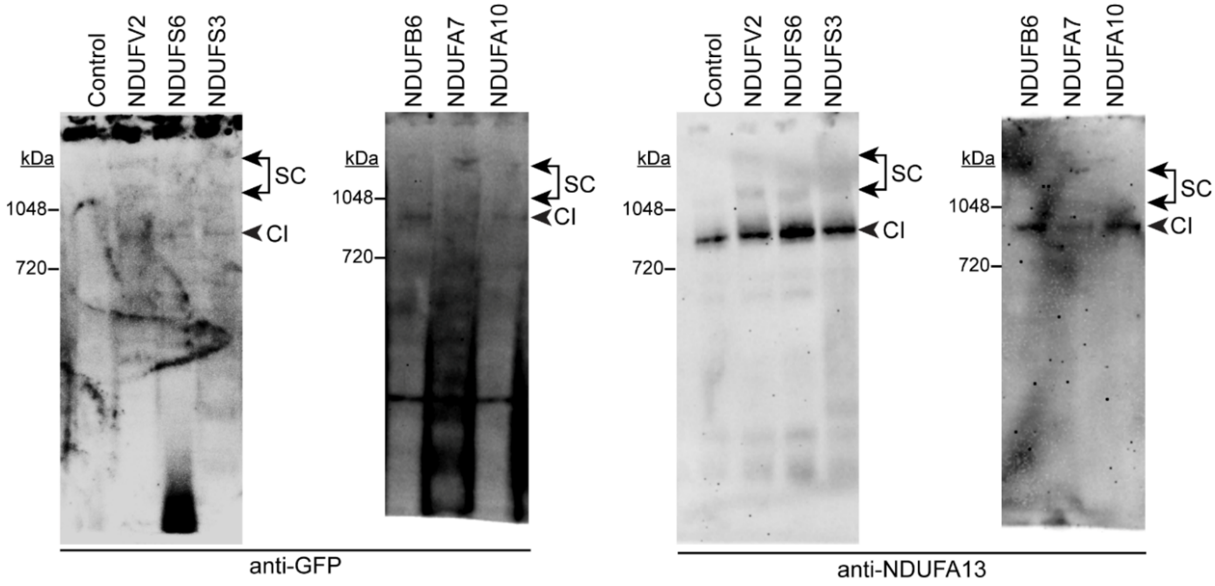

Supplementary Figure 21. BN-PAGE of mVenus-tagged NDUF subunits. Only those subunits that successfully targeted mitochondria were assayed by BN-PAGE. All integrate into Complex I (CI) or Complex I-containing supercomplexes (SC). Contrast and gamma have been adjusted to allow visualization of SC bands. Images are representative of 2 trials. Source data are provided as a Source Data file.

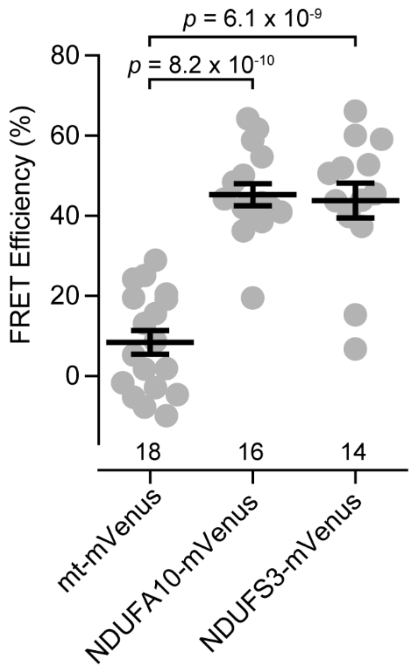

Supplementary Figure 22. Acceptor photobleaching confirms FRET interaction between MCU and Complex I subunits NDUFA10 and NDUFS3. Cells expressing MCU-mCerulean and the corresponding mVenus-tagged constructs were tested for donor dequenching after acceptor photobleaching using confocal microscopy. Summary data are presented as mean values ± SEM. Statistics: 1-way ANOVA followed by Bonferroni-corrected means comparisons. Source data are provided as a Source Data file.

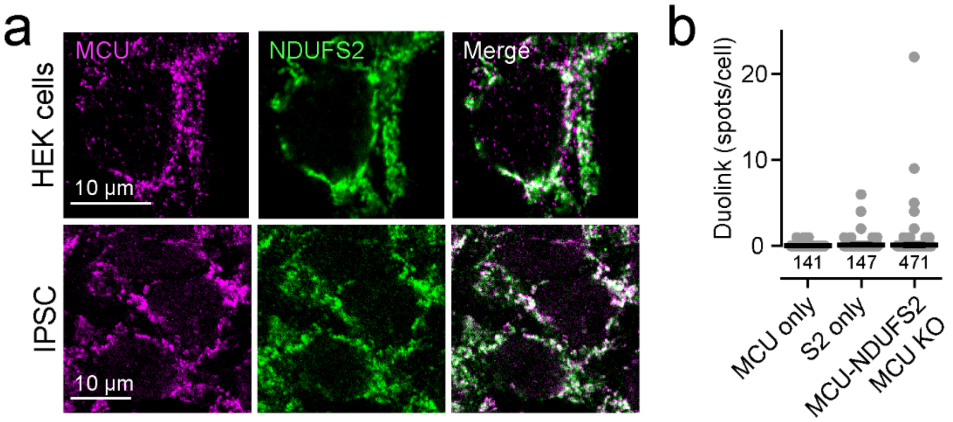

Supplementary Figure 23. Duolink validation. A. Rabbit anti-MCU and mouse anti-NDUFS2 both localize to mitochondria when used concurrently in HEK293T cells or IPSCs. **B.** Absence of substantial Duolink labeling in controls. 98%, 93%, and 98% of the cells had no Duolink spots when only MCU antibody was used (MCU only), only NDUFS2 antibody was used (S2 only), and both antibodies were used in MCU^{KO} cells (MCU-NDUFS2, MCU KO), respectively. Summary data are presented as mean values ± SEM. Statistics: 1-way ANOVA followed by Bonferroni-corrected means comparisons Source data are provided as a Source Data file.

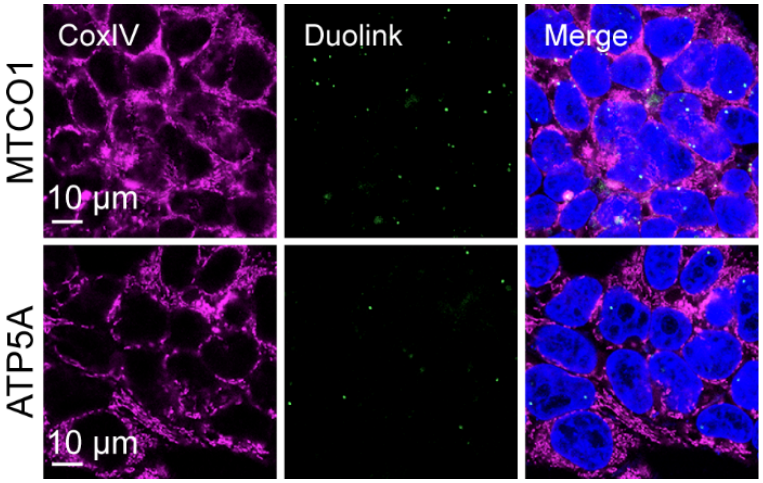

Supplementary Figure 24. Specificity of interaction with respirasome. Duolink assay using Complex IV component MTCO1 shows positive labeling. Much weaker Duolink signal when targeting the ATP synthase using ATP5A. Duolink data are quantified in Fig. 5c.

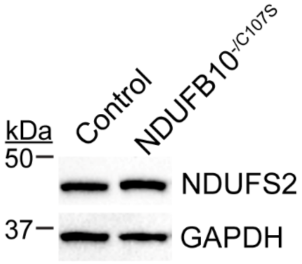

Supplementary Figure 25. Preserved NDUFS2 levels in patient-derived NDUFB10$^{-/C107S}$ IPSCs. Source data are provided as a Source Data file.

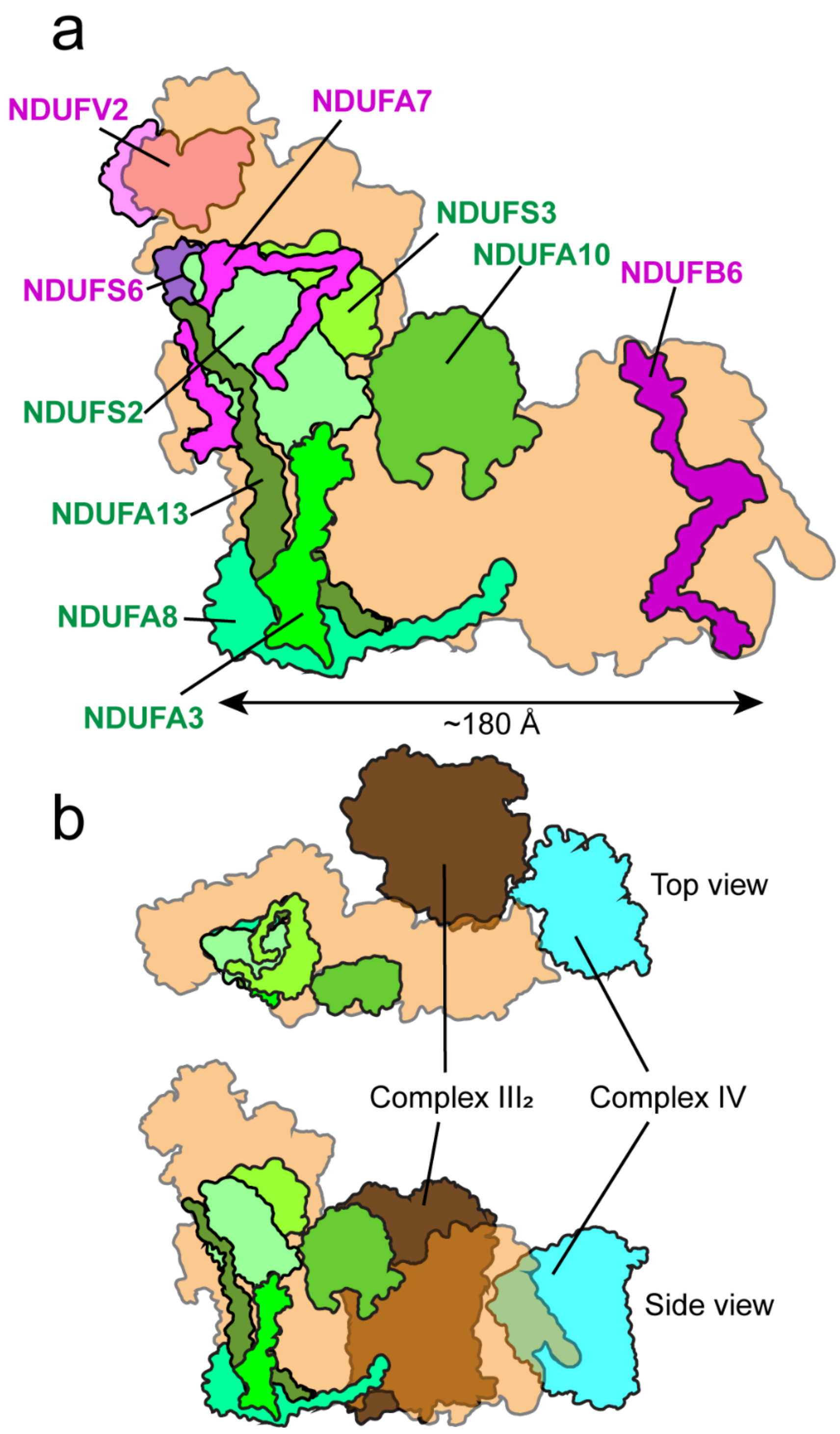

Supplementary Figure 26. Cartoon overview of MCU-Complex I interaction. **A.** Complex I subunits that interact with MCU are labeled green, while those that failed to interact are magenta. **B.** Top and side views of the respirasome (CI-CIII$_2$-CIV). The MCU-interacting subunits of Complex I form a surface cluster and are not sterically hindered in the respirasome by Complex III or IV, which are on the opposite sides.

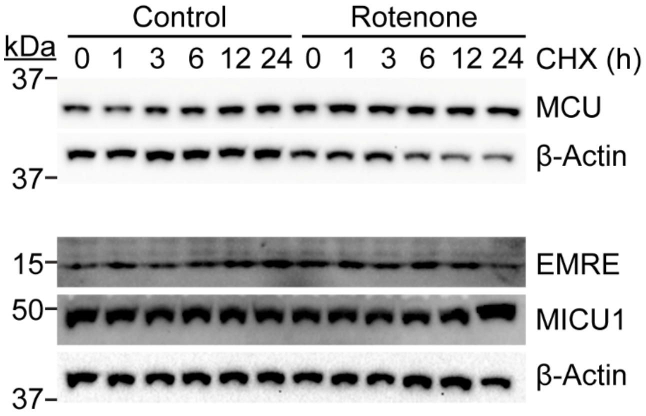

Supplementary Figure 27. Changes in uniporter subunit degradation. Protein levels of the uniporter subunits MCU, MICU1, and EMRE were evaluated over 24h of protein synthesis inhibition with 50 μg/mL cycloheximide treatment. A subtle persistence of MCU was noted relative to β-actin control, not seen for EMRE of MICU1. Images are representative of 3 trials. Source data are provided as a Source Data file.

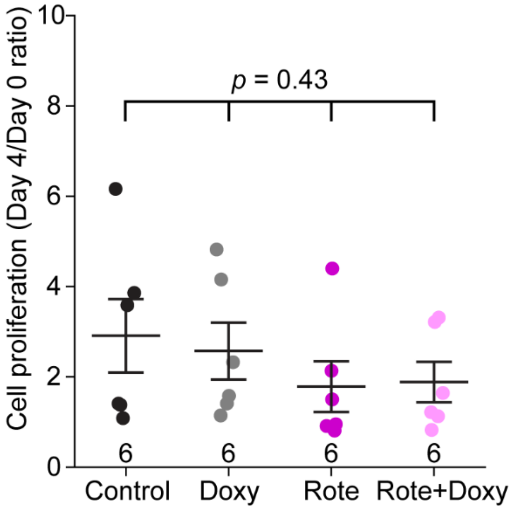

Supplementary Figure 28. Doxycycline addition did not substantially alter cell proliferation. Cells were grown for 4 days in low-FBS media containing 1 μg/mL doxycycline and/or 1 μM rotenone as indicated. Summary data are presented as mean values ± SEM. Statistics: Kruskal-Wallis test. Source data are provided as a Source Data file.

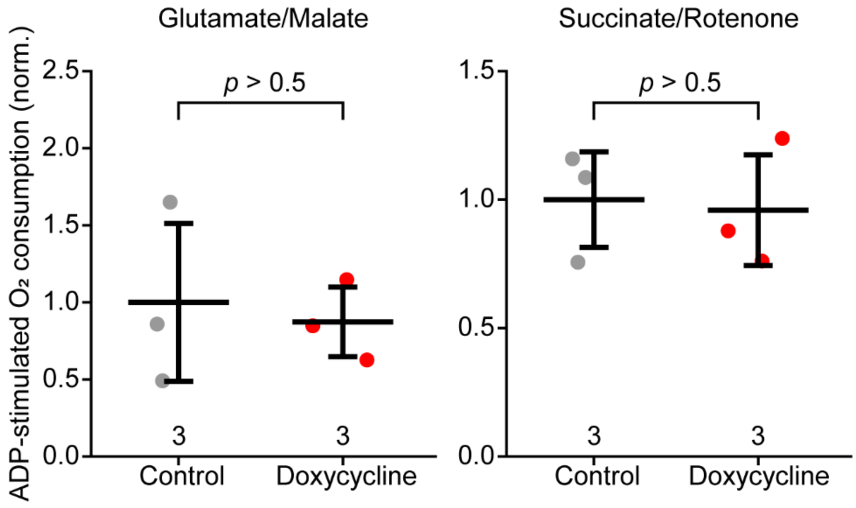

Supplementary Figure 29. Doxycycline did not affect cellular respiration. Cells were grown in 1 µg/mL doxycycline. Respiration with the substrates listed was measured in permeabilized cells. Summary data are presented as mean values ± SEM. Statistics: two-sided Mann-Whitney U test. Source data are provided as a Source Data file.

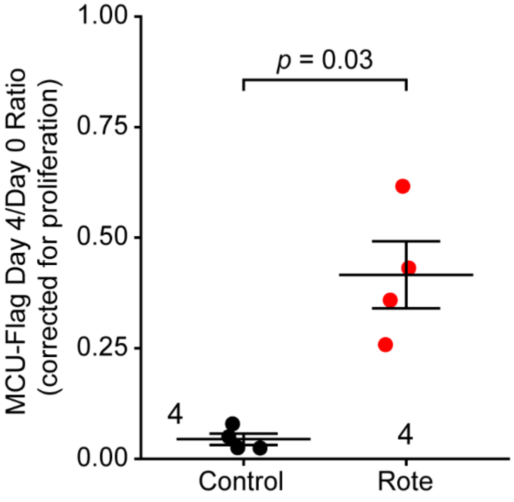

Supplementary Figure 30. Complex I impairment leads to reduced MCU degradation. MCU-Flag band density at day 0 and day 4 of doxycycline treatment was measured from Western blots. The ratio of band densities was corrected for cell proliferation in the corresponding treatment. Summary data are presented as mean values ± SEM. Statistics: two-sided Mann-Whitney U test. Source data are provided as a Source Data file.

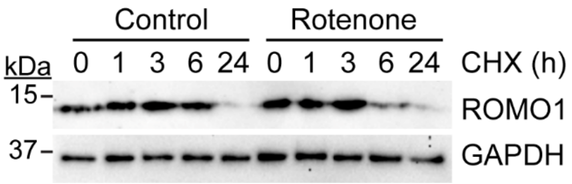

Supplementary Figure 31. ROMO1 stability is unaffected by rotenone treatment. Images are representative of 3 trials. Source data are provided as a Source Data file.

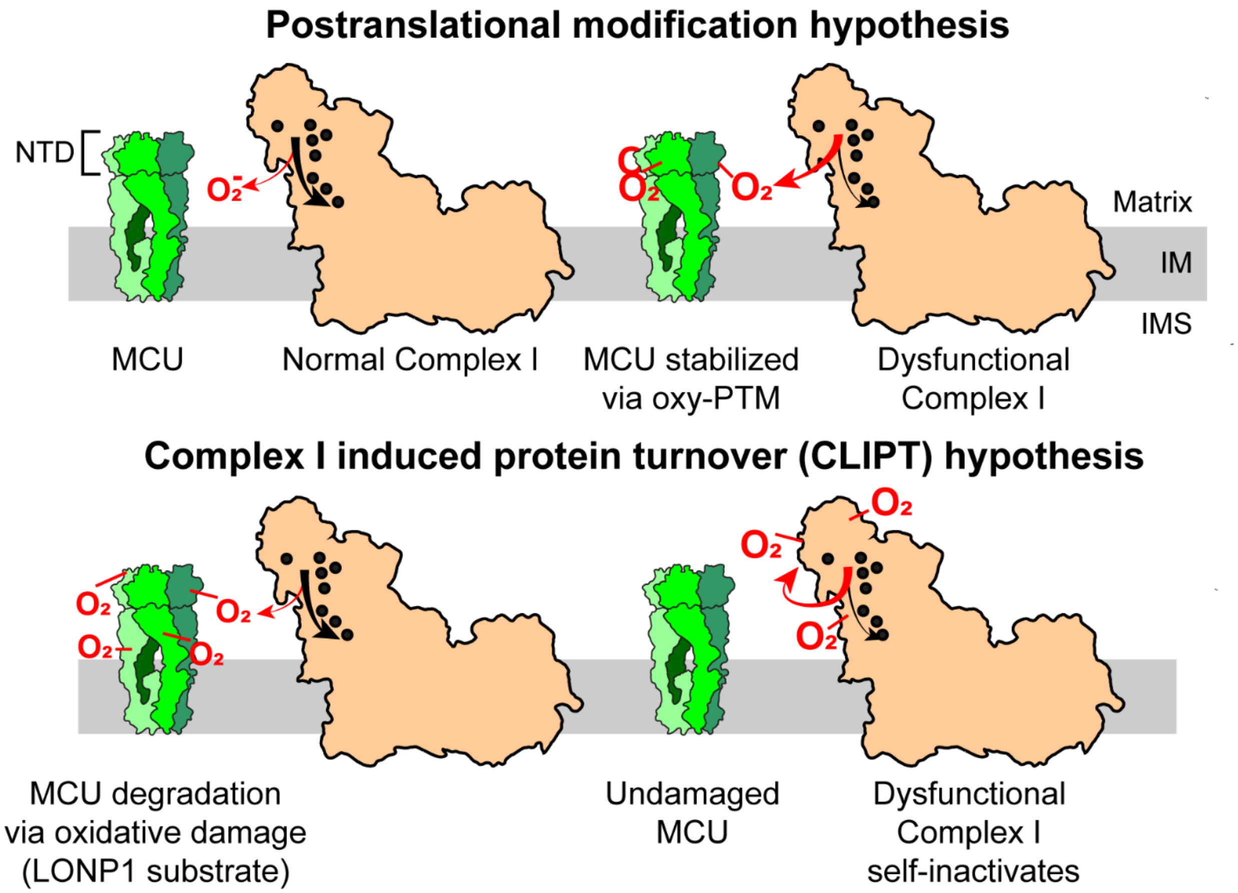

Supplementary Figure 32. Hypotheses for Complex I control of MCU stability. In the PTM hypothesis, ROS escaping Complex I produces specific oxidative modifications (oxy-PTM) on MCU that stabilize it. In the CLIPT hypothesis, under normal conditions MCU is turned over by quality control proteases due to constant promiscuous modifications from basal ROS leak from Complex I. Loss of Complex I activity, due to self-inactivating ROS burst or other mechanisms, prevents further MCU oxidative damage, thus stabilizing it.

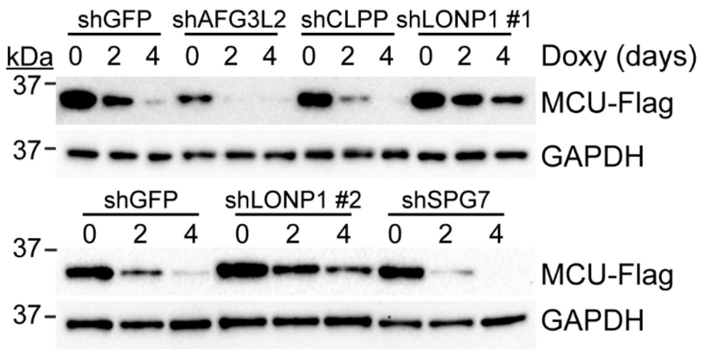

Supplementary Figure 33. MCU-Flag stability after RNAi of quality-control proteases reveals LONP1-mediated MCU degradation. shRNA produced 88-96% inhibition of the corresponding protease transcripts. Images are representative of 3 trials. Source data are provided as a Source Data file.

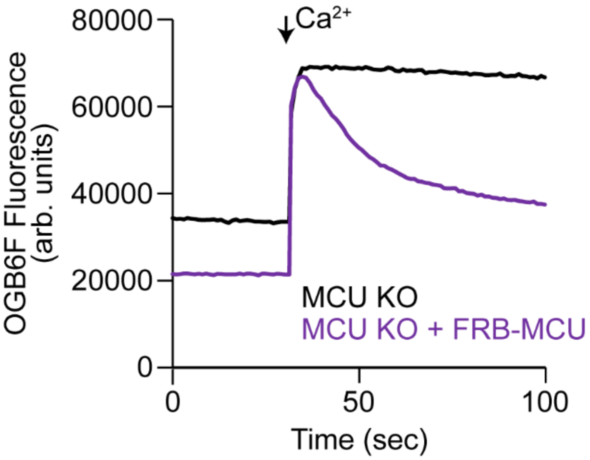

Supplementary Figure 34. Mitochondrial Ca^{2+} uptake is absent in digitonin-permeabilized MCU^{KO} cells but present after expression of FRB-MCU in these. Ca^{2+} fluorescence measured with Oregon Green BAPTA 6F (OGB6F). Arrow indicates 10 μM Ca^{2+} pulse.

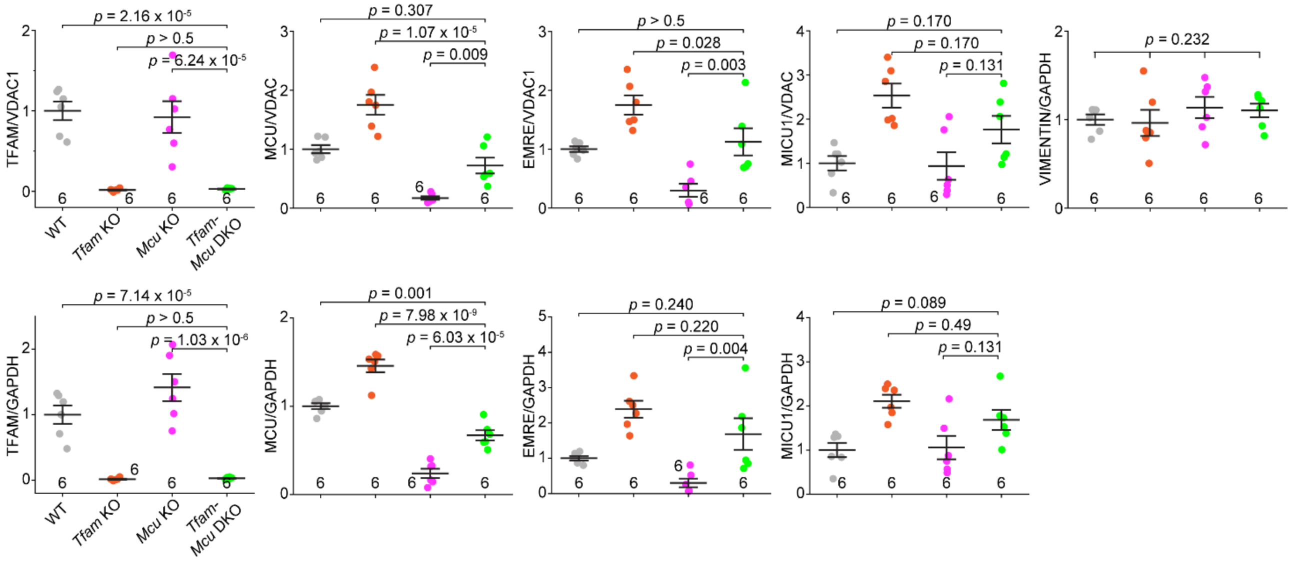

Supplementary Figure 35. Quantification of western blots in Figure 5a. The ratio of intensities relative to loading controls (GAPDH or VDAC1) were normalized to the average of the wild-type samples. Data collected from separate gels due to number of samples, each containing all four genotypes and processed in parallel. Summary data are presented as mean values ± SEM. Statistics: Kruskal-Wallis followed by Dunn's test with Bonferroni correction. Source data are provided as a Source Data file.

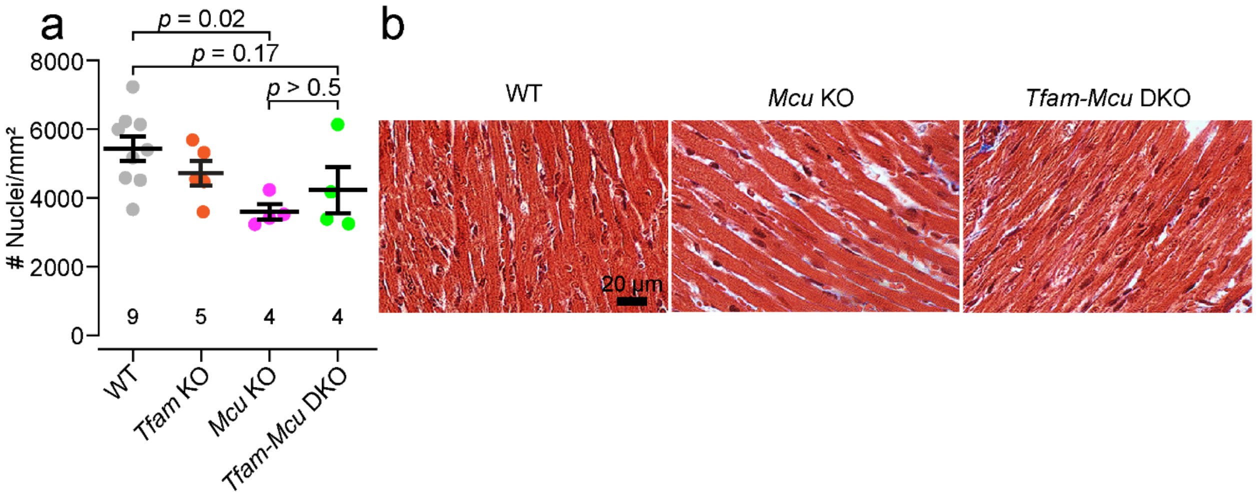

Supplementary Figure 36. *Tfam-Mcu* DKO mice do not have excess cells compared to *Mcu* KO. **A**. Automated analysis of nuclei density in immunohistological slices of cardiac tissue. N refers to number of mice studied. **B.** Masson's trichrome staining of mid-ventricular heart reveals no excess infiltrates or fibrosis in *Mcu* KO or *Tfam-Mcu* DKO mice. Summary data are presented as mean values ± SEM. Statistics: Kruskal-Wallis followed by Dunn's test with Bonferroni correction. Source data are provided as a Source Data file.

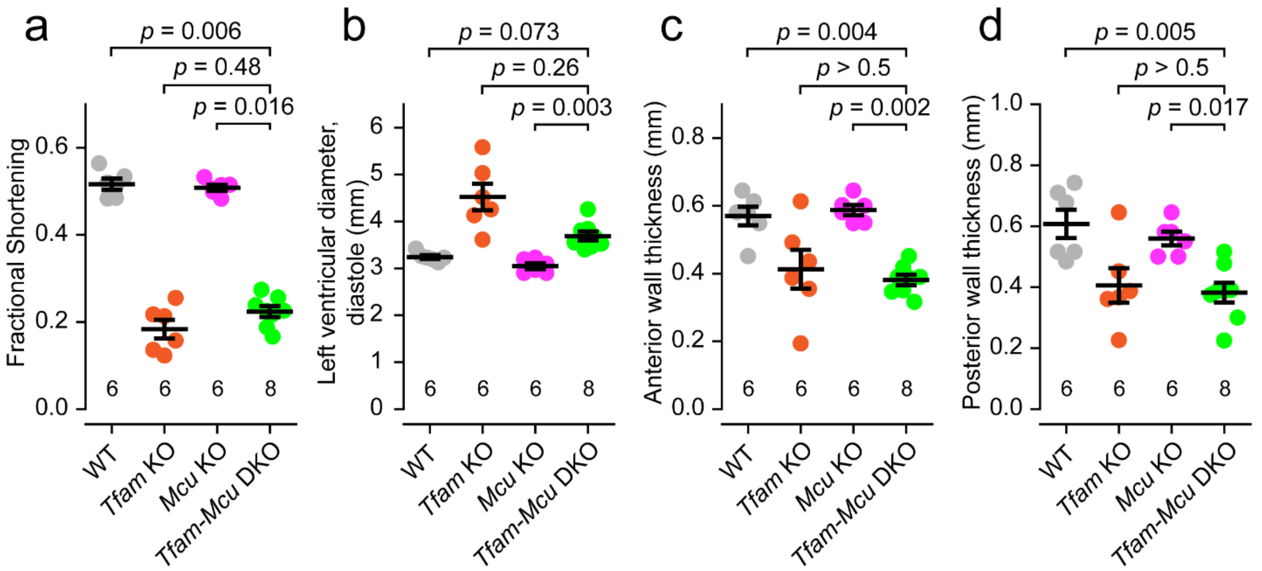

Supplementary Figure 37. *Tfam* KO and *Tfam-Mcu* DKO mice have a dilated cardiomyopathy. Echocardiographic parameters obtained from M-mode imaging. **C.** Fractional shortening. **D.** Left ventricular chamber diameter. **E, F.** Ventricular wall thicknesses. Summary data are presented as mean values ± SEM. Statistics: Kruskal-Wallis followed by Dunn's test with Bonferroni correction. Source data are provided as a Source Data file.

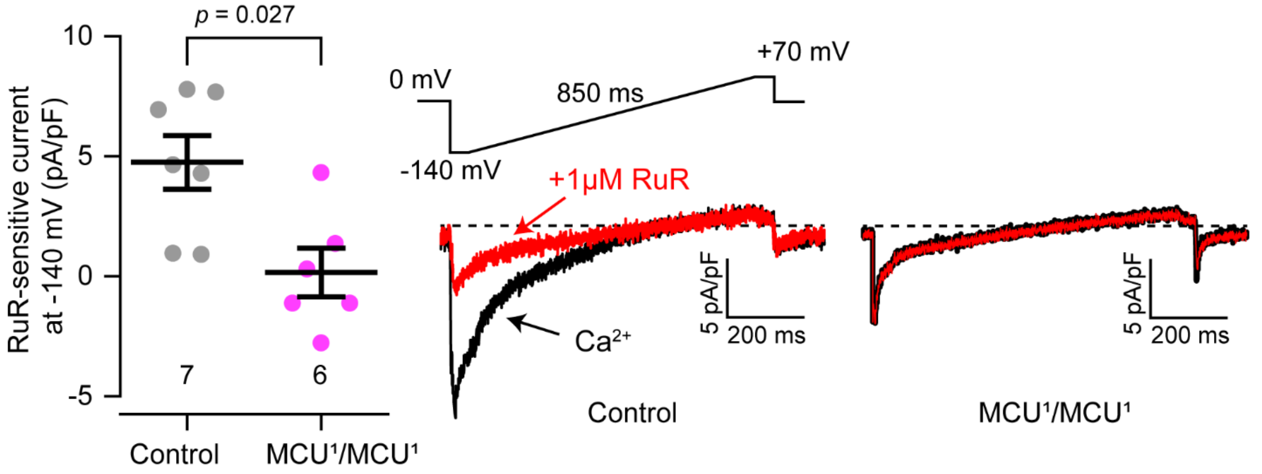

Supplementary Figure 38. No uniporter current is evident in *MCU*[1]/*MCU*[1] flies. Summary and exemplars for ruthenium-red sensitive I_{MiCa} in wild-type and *MCU*[1]/*MCU*[1] flies. Summary data are presented as mean values ± SEM. Statistics: two-sided Mann-Whitney U test. Source data are provided as a Source Data file.

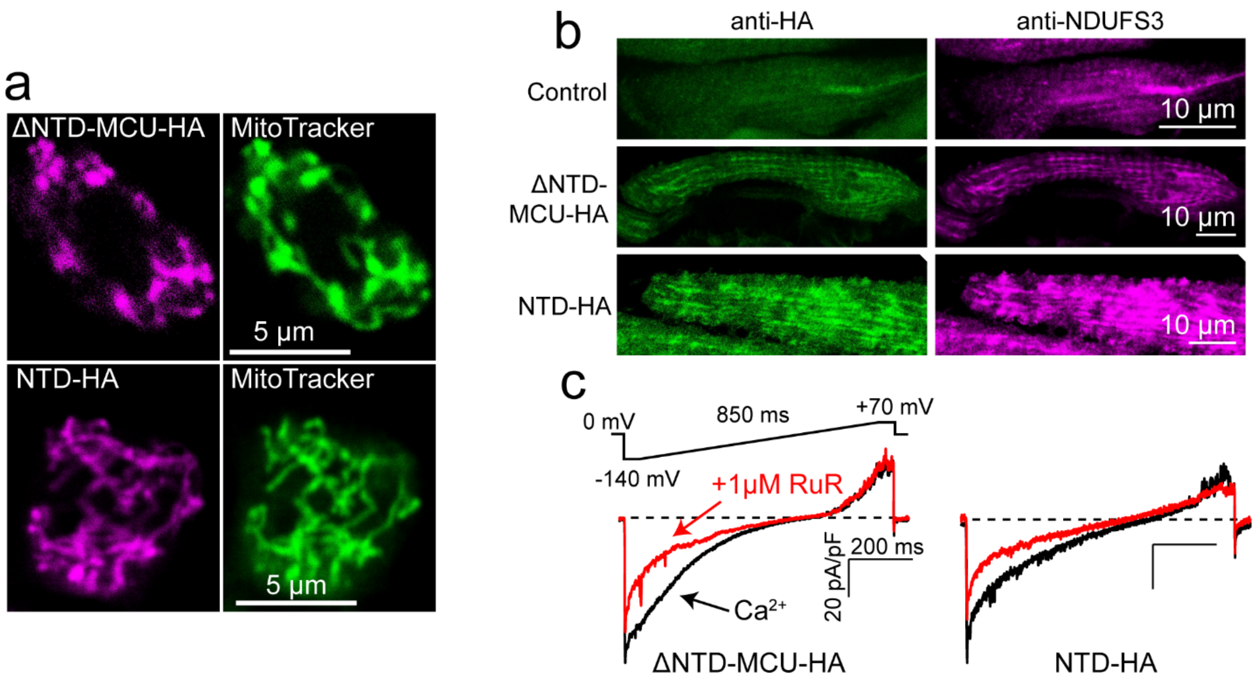

Supplementary Figure 39. Expression of *MCU* fragments. A. *Drosophila* S2 cell expressing ΔNTD-MCU and MCU NTD fragment. **B.** *Drosophila* flight muscle showing expression of ΔNTD-MCU and MCU NTD. **C.** Expression of ΔNTD-MCU produces ruthenium-red sensitive currents in *Drosophila* MCU knockout (MCU^1/MCU^1) mitochondria (left). Ca^{2+} current exemplar after expression of NTD-HA (right). (A, B) Images are representative of 2 trials.

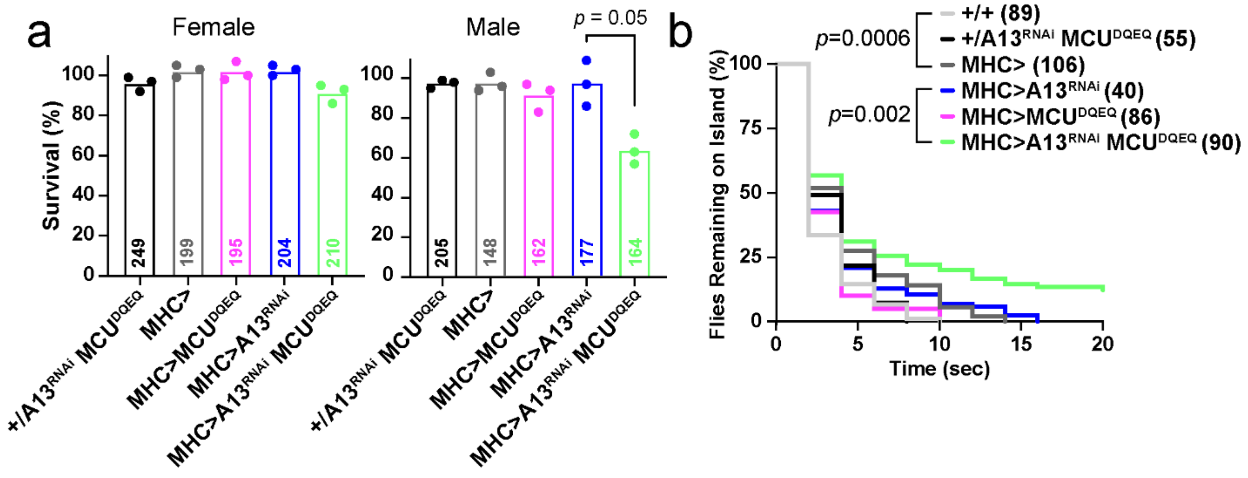

Supplementary Figure 40. Genetic interaction between MCU and NDUFA13 in *Drosophila*. A. Survival for $NDUFA13^{RNAi}$ and MCU^{DQEQ} crosses. **B.** Island assay. Statistics: (A) Fischer's Exact Test with Bonferroni correction; (B) Log-rank (Mantel-Cox) test with Bonferroni correction. Source data are provided as a Source Data file.

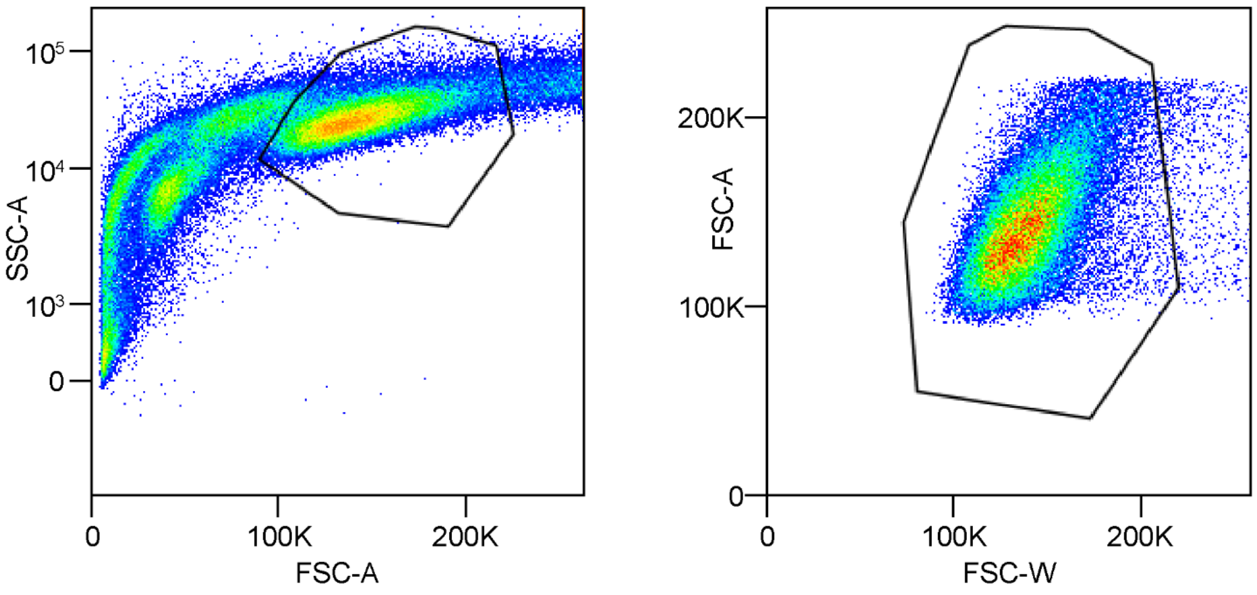

Supplementary Figure 41. Example gating strategy for flow cytometry. FSC-A, forward scatter-area; FSC-W, forward scatter-width; SSC-A, side scatter-area.
