# Supplementary material for: Mitochondrial calcium uniporter stabilization preserves energetic homeostasis during Complex I impairment
Source: Nat Commun. 2022 May 19;13:2769. doi: 10.1038/s41467-022-30236-4 (PMC9120069; doi:10.1038/s41467-022-30236-4)
Supplement: Supplementary file 3 — Description of Additional Supplementary Information [file 41467_2022_30236_MOESM3_ESM.pdf]

Description of Additional Supplementary Information

Supplementary Videos 1-9. Videos of fruit flies of the indicated genotype on the island assay.

1. MHC control: Flight muscle GAL4 expression driver
2. MHC with B10 RNAi: NDUFB10 RNAi in flight muscle
3. MCU[1]: MCU knockout
4. MHC with MCU[1] and B10[RNAi]: NDUFB10 RNAi in MCU knockout flies
5. MHC with B10[RNAi]-MCU[1]-MCU[WT}: MCU overexpressed in flies with NDUFB10 RNAi + MCU knockout.
6. MHC with MCU-DQEQ: flight muscle expression of dominant-negative MCU
7. MHC with B10[RNAi] and MCU-DQEQ: flight muscle expression of dominant-negative MCU in NDUFB10 RNAi flies.
8. MHC with B10[RNAi]-UAS-NTD: flight muscle expression of isolated NTD in NDUFB10 RNAi flies.
9. MHC with B10[RNAi]-MCU[1]-dNTD1: ΔNTD-MCU expression in flies with NDUFB10 RNAi + MCU knockout.
