# Supplementary material for: Mitochondrial calcium uniporter stabilization preserves energetic homeostasis during Complex I impairment
Source: Nat Commun. 2022 May 19;13:2769. doi: 10.1038/s41467-022-30236-4 (PMC9120069; doi:10.1038/s41467-022-30236-4)
Supplement: Supplementary file 13 — Reporting Summary [file 41467_2022_30236_MOESM13_ESM.pdf]

nature research

Corresponding author(s): Dipayan Chaudhuri
Last updated by author(s): Mar 22, 2022

Reporting Summary

Nature Research wishes to improve the reproducibility of the work that we publish. This form provides structure for consistency and transparency in reporting. For further information on Nature Research policies, see our Editorial Policies and the Editorial Policy Checklist.

Statistics

For all statistical analyses, confirm that the following items are present in the figure legend, table legend, main text, or Methods section.

n/a	Confirmed	
☐	☒	The exact sample size (*n*) for each experimental group/condition, given as a discrete number and unit of measurement
☐	☒	A statement on whether measurements were taken from distinct samples or whether the same sample was measured repeatedly
☐	☒	The statistical test(s) used AND whether they are one- or two-sided<br>*Only common tests should be described solely by name; describe more complex techniques in the Methods section.*
☒	☐	A description of all covariates tested
☐	☒	A description of any assumptions or corrections, such as tests of normality and adjustment for multiple comparisons
☐	☒	A full description of the statistical parameters including central tendency (e.g. means) or other basic estimates (e.g. regression coefficient) AND variation (e.g. standard deviation) or associated estimates of uncertainty (e.g. confidence intervals)
☐	☒	For null hypothesis testing, the test statistic (e.g. F, t, r) with confidence intervals, effect sizes, degrees of freedom and P value noted<br>*Give P values as exact values whenever suitable.*
☒	☐	For Bayesian analysis, information on the choice of priors and Markov chain Monte Carlo settings
☒	☐	For hierarchical and complex designs, identification of the appropriate level for tests and full reporting of outcomes
☒	☐	Estimates of effect sizes (e.g. Cohen's *d*, Pearson's *r*), indicating how they were calculated

Our web collection on statistics for biologists contains articles on many of the points above.

Software and code

Policy information about availability of computer code

Data collection	pClamp 10, Leica Application Suite X 3.5, MaxQuant 1.6, ImageLab 6.1, FACSDiva 6
Data analysis	OriginPro 9, Microsoft Excel 2016, FlowJo 10.6, GraphPad Prism 8, CellProfiler 3.1, Perseus 1.6, R 3.5

For manuscripts utilizing custom algorithms or software that are central to the research but not yet described in published literature, software must be made available to editors and reviewers. We strongly encourage code deposition in a community repository (e.g. GitHub). See the Nature Research guidelines for submitting code & software for further information.

Data

Policy information about availability of data

All manuscripts must include a data availability statement. This statement should provide the following information, where applicable:

- Accession codes, unique identifiers, or web links for publicly available datasets
- A list of figures that have associated raw data
- A description of any restrictions on data availability

Proteomics data have been deposited in PRIDE repository with the dataset identifier PXD018742

Field-specific reporting

Please select the one below that is the best fit for your research. If you are not sure, read the appropriate sections before making your selection.

☒ Life sciences ☐ Behavioural & social sciences ☐ Ecological, evolutionary & environmental sciences

For a reference copy of the document with all sections, see nature.com/documents/nr-reporting-summary-flat.pdf

Life sciences study design

All studies must disclose on these points even when the disclosure is negative.

Sample size	Calculations using Student's t-test or 1-way ANOVA for 2-4 groups, assuming power of 0.8-0.9, alpha < 0.05, and coefficient of variation of 20-30% produce sample size estimates in the 5-15 range, so our experiments were designed to obtain sample sizes in this range. For electrophysiology, data was collected on at least three separate days for each experiment, aiming for 5-15 mitoplasts per group, which was based on the effect sizes seen in our prior publication (Sommakia et. al. PMID 28962857).
Data exclusions	Only inwardly-rectifying currents and/or ruthenium-red sensitive currents were analyzed for measuring uniporter currents via electrophysiology.
Replication	Fly viability and island assay data was performed on at least three separate crosses. FRET assays were repeated 7 times over several months. Western blots were repeated three times. More than one litter was used for all mouse studies. We also replicated findings using different methodologies (e.g. coIP and FRET and Duolink for Complex I-MCU interaction), or different mutants (e.g. NDUFB10 v NDUFA13 RNAi for flies, FOXRED1-KO v NDUFB10-KO for electrophysiology). Other experiments were repeated 2-3 times successfully.
Randomization	No randomization was used. Drug or vehicle were applied to cells under the same conditions. Other tests compared identical protocols between mutant and control groups.
Blinding	Blinding was not performed as we were not allocating treatments. All analyses were performed identically between groups.

Reporting for specific materials, systems and methods

We require information from authors about some types of materials, experimental systems and methods used in many studies. Here, indicate whether each material, system or method listed is relevant to your study. If you are not sure if a list item applies to your research, read the appropriate section before selecting a response.

Materials & experimental systems

n/a	Involved in the study	
☐	☒	Antibodies
☐	☒	Eukaryotic cell lines
☒	☐	Palaeontology and archaeology
☐	☒	Animals and other organisms
☒	☐	Human research participants
☒	☐	Clinical data
☒	☐	Dual use research of concern

Methods

n/a	Involved in the study	
☒	☐	ChIP-seq
☐	☒	Flow cytometry
☒	☐	MRI-based neuroimaging

Antibodies

Antibodies used	ATP5F1 (ab117991, Abcam, Lot # GR63355-5), β-Actin (ab8224, Abcam, GR3259037-3), COX IV-Alexa Fluor 488 (4853S , Cell Signaling Tech [CST], 7), EMRE (A300-BL19208, Bethyl, 141204), FLAG HRP (A8592, Sigma, SLBV3799), FLAG magnetic beads (M8823, Sigma), FOXRED1 (sc-377264, Santa Cruz, I2012), GAPDH (2118S, CST, 14), GFP (ab290, Abcam, GR331575-1), goat anti-mouse Alexa Fluor 555 (A21422, ThermoFisher, 2090527), goat anti-rabbit Alexa Fluor 488 (A32731, ThermoFisher, UD282059), HA (3724S, CST, 9), HA HRP (12013819001, Sigma), MCU (14997S, CST, 1), MICU1 (12524S, CST, 1:1000), MTCO1 (ab14705, Abcam, GR3228460-1), NDUFA13 (ab110240, Abcam, GR3199442-2), NDUFB10 (ab196019, Abcam, GR195930-2), NDUFS2 (ab110249, Abcam, GR3238733-1), NDUFS3 (ab177471, Abcam, GR3267320-4), NDUFS4 (ab137064, Abcam, GR268471-4), Oct4 (ab19857, Abcam, GR3266058-8), ROMO1 (TA505580, Origene, F001), Sox2 (5024, CST, 1), TOM20 (42406S, CST), VDAC1 (ab14734, Abcam, GR3296736-21), Vimentin (ab92547, Abcam), ZO-1 (33-9100, ThermoFisher, UG286808).
Validation	The following antibodies have been validated by the manufacturer for the Western blot, immunofluorescence, and/or affinity purification applications used in this work. The catalog number and number of citations referenced at the manufacturer's or CiteAb website are included in parentheses. Anti-β-Actin (ab8224, 268 citations, e.g. PMID 32686675), Anti-ATP5F1 (ab117991, 5 citations, e.g. PMID 32877677), Anti-COX IV-Alexa 488 (4853S, 3 citations, e.g. PMID 33526710), Anti-FLAG HRP (A8592-.2MG, 778 citations, e.g. PMID 33712617), Anti-FLAG mag beads (M8823-1ML, 147 citations, e.g. PMID 33203880), Anti-FOXRED1 (sc-377264, 1 citation, e.g. PMID 23915000), Anti-GAPDH (2118S, 2547 citations, e.g. PMID 33147445), goat anti-mouse Alexa Fluor 555 (A21422, 711 citations, PMID 30339698), goat anti-rabbit Alexa Fluor 488 (A32731, 419 citations, e.g. PMID 34462441), Anti-HA HRP

(12013819001, 2 citations, e.g. PMID: 33087347), Anti-MCU (14997S, 15 citations, e.g. PMID 26119742), Anti-MTCO1 (ab14705, 280 citations, PMID 26785948), Anti-NDUFA13/GRIM19 (ab110240, 30 citations, e.g. PMID 32402267), Anti-NDUFB10 (ab196019, 3 citations, e.g. PMID 34021238), Anti-NDUFS2 (ab110249, 6 citations, e.g. PMID 28424233), Anti-NDUFS3 (ab177471, 3 citations, e.g. PMID 33087354), Anti-NDUFS4 (ab137064, 1 citation, e.g. PMID 31685978), Anti-ROMO1 (TA505580, 8 citations, e.g. PMID 30598479), Anti-OCT4 (ab19857, 338 citations, e.g. PMID 32671061), Anti-SOX2 (5024, 13 citations, e.g. PMID 25184684), Anti-TOM20 (42406S, 134 citations, e.g. PMID 32102993), Anti-Vimentin (ab92547, 808 citations, e.g. PMID 32404936), Anti-ZO1 (33-9100, 790 citations, e.g. PMID 33169669), Anti-HA (3724S, 544 citations, e.g. PMID 32948771), Anti-VDAC1 (ab14734, 425 citations, e.g. PMID 30201738). The anti-MCU antibody has been knockout validated (PMID 26119742) and validated for imaging (PMID 27637331). The anti-NDUFS2 has been knockout validated by the manufacturer. The anti-NDUFA13 antibody has been knockout validated (PMID 29078279). The anti-EMRE antibody has been knockout validated (PMID 24231807). Anti-ATP5F1 has been used in HEK293 cells (PMID 32877677) and we further validate it for confocal imaging studies here. We further knockout validate the NDUFB10 antibody here for Western blot , validate the anti-MCU and anti-NDUFS2 antibodies for the Duolink application, and the anti-GFP for BN-PAGE.

Eukaryotic cell lines

Policy information about cell lines

Cell line source(s)	HEK293T cell line (gift of David Clapham), patient-derived induced pluripotent stem cells
Authentication	Authentication was by short tandem repeat analysis. Validation of mutation was by sequencing and Western blot.
Mycoplasma contamination	Cell lines tested negative for mycoplasma contamination
Commonly misidentified lines (See ICLAC register)	No

Animals and other organisms

Policy information about studies involving animals; ARRIVE guidelines recommended for reporting animal research

Laboratory animals	Drosophila melanogaster (Berlin strain) were of both sexes and aged 3-6 days old. Mice were C57BL/6J of both sexes and aged P10-P14. Mice are housed in 12h each light/dark cycles, at 21 degrees Celsius, at 26% humidity.
Wild animals	None
Field-collected samples	None
Ethics oversight	We have University of Utah IACUC approval (#19-01004) for mouse studies. IACUC approval is not required for experiments involving Drosophila.

Note that full information on the approval of the study protocol must also be provided in the manuscript.

Flow Cytometry

Plots

Confirm that:

☒ The axis labels state the marker and fluorochrome used (e.g. CD4-FITC).

☒ The axis scales are clearly visible. Include numbers along axes only for bottom left plot of group (a 'group' is an analysis of identical markers).

☒ All plots are contour plots with outliers or pseudocolor plots.

☒ A numerical value for number of cells or percentage (with statistics) is provided.

Methodology

Sample preparation	HEK293T cells grown in culture and transfected with fluorescent constructs (mitoSoNar, mitoGCaMP6m-mCherry, mVenus- or mCerulean-tagged constructs) or incubated with fluorescent sensors (MitoSOX) were dispersed in phosphate-buffered saline and fluorescence was measured. No tissue processing was performed.
Instrument	BD FACSCanto
Software	BD FACSDiva 6
Cell population abundance	We analyzed single live cells that typically represented >50% of the collected population.
Gating strategy	We gated live cells by looking for the population that clustered around forward scatter area 100K-200K and side scatter area 10K-100K.

☒ Tick this box to confirm that a figure exemplifying the gating strategy is provided in the Supplementary Information.
